# A Three-Genes Signature Predicting Colorectal Cancer Relapse Reveals LEMD1 Promoting CRC Cells Migration by RhoA/ROCK1 Signaling Pathway

**DOI:** 10.3389/fonc.2022.823696

**Published:** 2022-05-10

**Authors:** Hui Zhang, Chenxin Xu, Feng Jiang, Jifeng Feng

**Affiliations:** ^1^Department of General Surgery, The Affiliated Cancer Hospital of Nanjing Medical University, Jiangsu Cancer Hospital, Jiangsu Institute of Cancer Research, Nanjing, China; ^2^Research Center for Clinical Oncology, The Affiliated Cancer Hospital of Nanjing Medical University, Jiangsu Cancer Hospital, Jiangsu Institute of Cancer Research, Nanjing, China; ^3^Jiangsu Key Laboratory of Molecular and Translational Cancer Research, Jiangsu Cancer Hospital, Jiangsu Institute of Cancer Research, The Affiliated Cancer Hospital of Nanjing Medical University, Nanjing, China

**Keywords:** LEMD1, relapse model, RhoA, EMT, colorectal cancer, metastasis

## Abstract

**Objective:**

Colorectal cancer (CRC) patients that experience early relapse consistently exhibit poor survival. However, no effective approach has been developed for the diagnosis and prognosis prediction of postoperative relapsed CRC.

**Methods:**

Multiple datasets from the GEO database and TCGA database were utilized for bioinformatics analysis. WGCNA analyses and RRA analysis were performed to identify key genes. The COX/Lasso regression model was used to construct the recurrence model. Subsequent *in vitro* experiments further validated the potential role of the hub genes in CRC.

**Results:**

A comprehensive analysis was performed on multiple CRC datasets and a CRC recurrence model was constructed containing LEMD1, SERPINE1, and SIAE. After further validation in two independent databases, we selected LEMD1 for *in vitro* experiments and found that LEMD1 could regulate CRC cell proliferation, migration, invasion, and promote EMT transition. The Rho-GTPase pulldown experiments further indicated that LEMD1 could affect RhoA activity and regulate cytoskeletal dynamics. Finally, we demonstrated that LEMD1 promoted CRC cell migration through the RhoA/ROCK1 signaling pathway.

**Conclusions:**

In this study, a CRC relapse model consisting of LEMD1, SERPINE1, and SIAE was constructed by comprehensive analysis of multiple CRC datasets. LEMD1 could promote CRC cell migration through the RhoA/ROCK signaling pathway.

## Introduction

Colorectal cancer (CRC), which ranks third in morbidity and mortality among all types of cancers, is one of the most common gastrointestinal malignancies in the world (1). Postoperative recurrence is considered a common disease event that severely affects prognosis. Although carcinoembryonic antigen (CEA) is commonly used as a clinical indicator of CRC, it still has a limitation of low precision and specificity in assessing postoperative recurrence (2). Application of other molecular markers such as loss of heterozygosity, p53 mutations, and microsatellite instability as prognostic markers requires further clinical evaluation (3).

Tumor metastasis involves a multistep biological process known as invasion and metastasis cascade (4). In this process, tumor cells lose their epithelial phenotype and acquire a more mobile mesenchymal phenotype called epithelial-mesenchymal transition (EMT) (5). Currently, targeted therapies for these processes are limited.

RhoA is one of the most studied members of the Rho family of small GTPases. It activates downstream effector molecules in its GTP-activated form, thereby affecting the cytoskeleton, cell adhesion, cell migration, and EMT processes (6). Previous studies have shown that abnormal expression of RhoA is common in a variety of tumors, including CRC, and activation of RhoA was associated with tumor metastasis (7).

LEMD1 (LEM domain containing 1) belongs to cancer testicular antigen (CTA), which is only expressed in normal testes, and oncogenic CTA is the target of cancer immunotherapy (8). Previous studies have shown that LEMD1 is abnormally expressed in oral squamous cell carcinoma (9), colorectal cancer (CRC) (10), prostate cancer (11), and anaplastic large cell lymphoma (12). Zhang et al. demonstrated that LEMD1 can promote the proliferation of gastric cancer cells through the PI3K/Akt signaling pathway (13). Takeda et al. found that LEMD1 promoted the adhesion of CRC stem cells (14). However, the mechanism of LEMD1 in CRC remains to be further elucidated.

In this study, we performed a comprehensive analysis on multiple CRC datasets and then constructed a CRC relapse model consisting of LEMD1, SERPINE1, and SIAE. After validation of this model, we selected LEMD1 for *in vitro* experiments and found that LEMD1 could regulate CRC cell proliferation, migration, invasion, and promote EMT. Further Rho-GTPase pull-down experiments proved that LEMD1 could affect RhoA activity and regulate cytoskeletal dynamics. Finally, we demonstrated that LEMD1 promoted the migration of CRC cells through the RhoA/ROCK signaling pathway.

## Materials and Methods

### Preprocessing of Microarray Data

Raw microarray CRC datasets were obtained from the GEO database (https://www.ncbi.nlm.nih.gov/geo/) and TCGA database (https://portal.gdc.cancer.gov/). In GEO database, we screened the datasets according to the keyword “colon cancer”, and the selected datasets should contain cancer and adjacent information (sample size should be greater than 25 cases) or prognostic information (sample size should be greater than 90 cases). Finally, six datasets (GSE21510, GSE113513, GSE74602, GSE24550, GSE89076, and GSE110224) from the GEO database containing colon cancer and adjacent tissues were used to detect differentially expressed genes. The GSE33313, GSE39582 and TCGA datasets were used to construct and validate colon cancer prognostic models. Data were normalized using Robust Multichip Average (15). All probes were mapped based on their own EntrezGeneID. When multiple probes were mapped to the same EntrezGeneID, the mean value was used to represent its average expression level.

### Construction of the CRC Relapse Model

**Figure 1:**
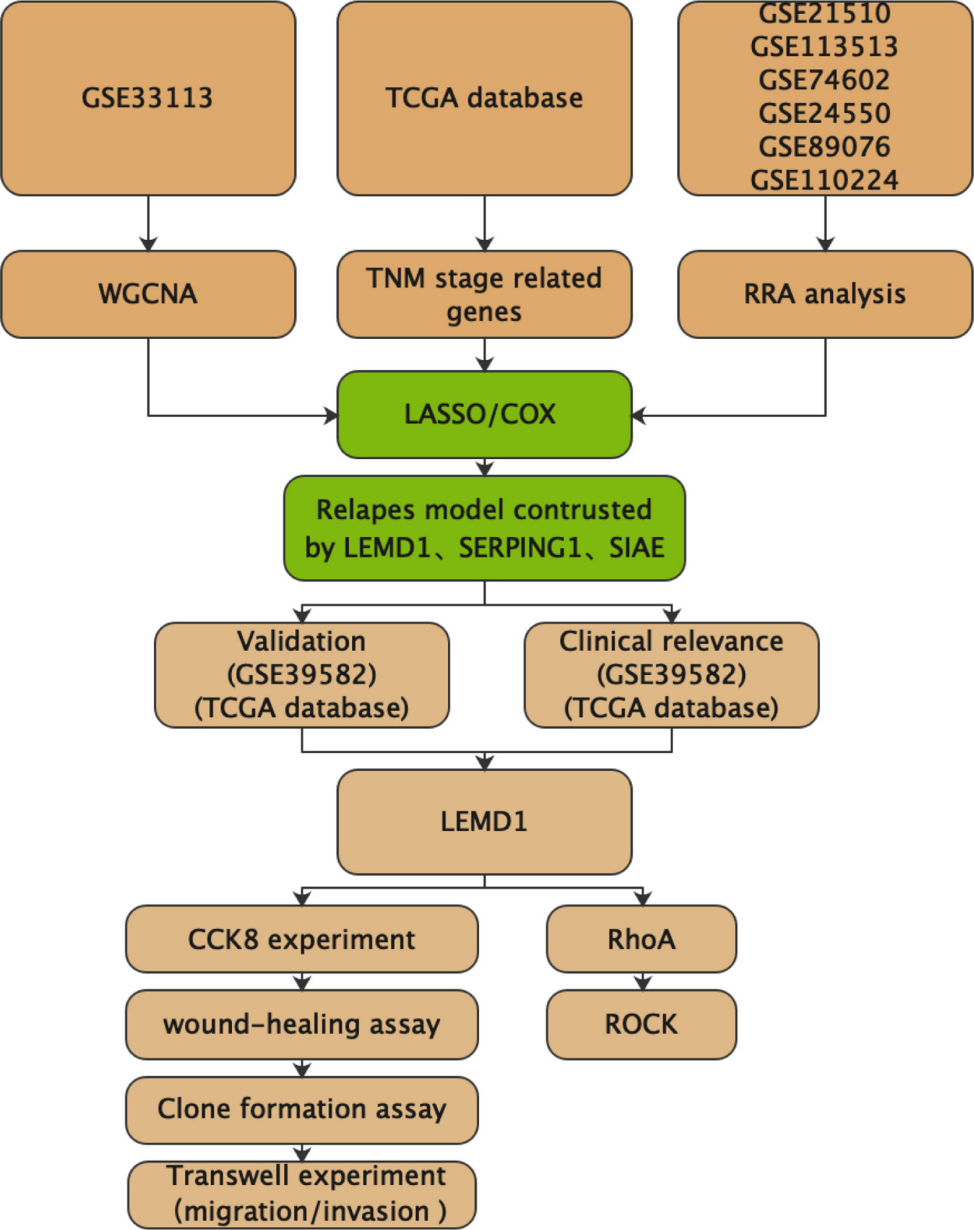
Workflow for data generation and analysis.

To select key genes for the construction of the recurrence model, we used WGCNA analysis, RRA analysis, and TNM staging differential expression analysis (**Figure 1**). The COX/Lasso regression model was used to construct the recurrence model. The GSE39582 and TCGA databases were used to further validate the diagnostic value of the model.

#### ① Weighted Correlation Network Analysis (WGCNA)

WGCNA is a systematic biological method used to describe the pattern of gene correlation between different samples. It can identify highly synergistic gene modules and candidate biomarkers based on the intrinsic connections of gene networks (16, 17). We selected the GSE33113 dataset to construct a gene co-expression network and selected the top 20% among the 23,494 genes. The minimum number of module genes was defined as 40. The correlation between modules and clinicopathological characteristics, including age, sex, and disease recurrence at the end of the observation period, was evaluated.

#### ② Robust-Rank Aggregation Analysis

Robust-Rank Aggregation (RRA) was used to integrate gene lists into multiple sets of chip data (18). This method avoids cross-platform standardization and the limitation of the number of samples for each chip, which is significant to evaluate differential gene expression profiles (19, 20). We performed a comprehensive RRA analysis using the “limma” and “RobustRankAggreg” packages in six datasets (GSE21510, GSE113513, GSE74602, GSE24550, GSE89076, and GSE110224). A P-value <0.05 and Log |FC| ≥ 1 were considered statistically significant.

#### ③ COX/LASSO Regression Model

The COX/LASSO regression model was performed using the GSE33113 dataset, which included 90 cases of postoperative recurrence of colon cancer. Using COX/LASSO regression analysis, we identified a panel of genes and constructed a multigene-based classifier to predict early relapse in patients with CRC in GSE33113. With a specific risk score formula, patients from different sets were divided into high- and low-risk groups using the median risk score as the cut-off point. Survival differences between low- and high-risk groups in each set were evaluated using the Kaplan–Meier estimate and compared using the log rank test. Multivariate Cox regression analysis and data stratification analysis were performed to assess the independent prognostic role of the risk score to predict the RFS. Time-dependent receiver-operating characteristic (ROC) analysis was used to investigate the prognostic or predictive accuracy of each feature and signature. All statistical analyses were performed with R (version 4.0.3, www.r-project.org). All statistical tests were two-sided and P-values < 0.05 were considered statistically significant.

### Cell Culture

Human CRC cell lines SW480, HCT116, SW620, LOVO, DLD1, HFC, and NCM460 were purchased from the American Type Culture Collection (Manassas, Virginia, USA). Cells were cultured at 37°C in a 5% CO_2_ incubator in Dulbecco’s modified Eagle’s medium (KeyGEN BioTECH, Jiangsu, China) with 10% fetal bovine serum (Gibco, USA).

### shRNA Knockdown and LEMD1-Overexpression

LEMD1 shRNAs for LEMD1 were constructed and cloned into GV248-GFP-Puro (Genechem Biotech, Inc.). Transduced cells were selected in 10 mg/mL of puromycin and then were sorted by flow cytometry. LEMD1 overexpressing and LEMD1 knockdown cells were cultured and maintained in 2 mg/mL of puromycin. The target sequences for shLEMD1 were as follows: LEMD1 shRNA (sh LEMD1): 5′-CAGAATCACATATGGGACTAT-3′.

LEMD1-overexpression was conducted as previously described (21). Briefly, Lentivirus-LEMD1 (LV-LEMD1) was constructed in the lentivirus vector GV492 (Genechem Biotech Inc, Shanghai, China), while the lentivirus vector GV492 was adopted as a control. LV-LEMD1 was then packaged in 293T cells. Subsequently, the supernatants that contained viruses were infected with CRC cells for 16 h. After infection, stable clones were selected with 2 mg/mL puromycin (Sigma-Aldrich). Infection efficiency was validated using real-time RT–PCR or Western blot assays.

### RNA Extraction, Reverse Transcription

Tissue and blood RNA were extracted using the Tissue RNA Kit (OMEGA Bio-tek, R6688-01, USA) and the Blood RNA Kit (OMEGA bio-tek, R6814-01C, USA) according to the manufacturers’ instructions. TRIzol reagent (Invitrogen) was utilized to extract RNA from cultured cells according to the manufacturer’s instructions. A ratio of (A260)/(A280) is an indication of nucleic acid purity. A value greater than 1.8 indicated > 90% nucleic acid purity. For reverse transcription, 1 μg RNAs were inversely transcribed into 20 μL cDNA with a reverse transcription kit (Takara, Dalian, China). The relative expression of LEMD1 was determined in three independent experiments and normalized using the 2^−ΔΔCt^ method relative to GAPDH. The primers used in this experiment are shown in **DATA S1**.

### Clone Formation Assay

The cell suspension was diluted at a gradient comprising multiples of 100, 200, and 500 cells per six-well plate, inoculated in a Petri dish, and gently rotated to evenly disperse the cells. Cells were cultured for 2 weeks at 37°C in a 5% CO_2_ environment and then fixed in 4% paraformaldehyde for 20 min and then subjected to crystal violet staining for 30 min.

### Cell Cycle Experiments

Cell counting and cell cycle experiments were performed according to instructions of Cell cycle detection Kit (KeyGEN BioTECH, KGA512).

### CCK8, Transwell, Wound-Healing Assays, Cell Culture, and qRT-PCR

CCK8, Transwell, wound healing assays, cell culture, and qRT-PCR were performed as described previously (22).

### Western Blot

A 200 μL volume of lysis buffer (RIPA: PMSF = 100:1) was added to each well of a 6-well plate and lysed on ice for 30 min. After centrifugation at 1200 rps/s at 4°C for 20 min, the protein concentration was measured using the BCA method. Immunoblotting was performed as previously described (23). The antibodies were as follows: anti-LEMD1(abcam, #Ab201206), GAPDH (CST, #5174), E-cadherin (CST, #14472), Vimentin (CST, #5741), and Vinculin (Abcam, #Ab129002).

### Rho GTPases Pulldown Assay

Rho GTPase activity detection was performed using a Pull-Down Activation Assay Kit (Cytoskeleton, USA).

### Statistical Analysis

The qRT-PCR results are calculated using 2^-△△^ and then transformed according to log2^(X + 1)^. In assays such as migration, proliferation, or invasion, data were analyzed using Student’s t test (for two samples) or one-way analysis of variance (ANOVA), for more than two samples. The Wilcoxon rank sum test was used to compare the two groups of clinical specimens. The signed rank test was used for the comparison of paired samples. The Kruskal–Wallis test was used to compare multiple specimens. Pearson’s correlation was performed to compare the relationship between the risk score and clinical data. P <0.05 was considered statistically significant. Decision Curve Analysis (DCA) was used to evaluate risk score and mismatch repair (MMR) as predictors of chemotherapy in patients with stage II colon cancer (24).

## Results

### Screening for Hub Genes

**Figure 2:**
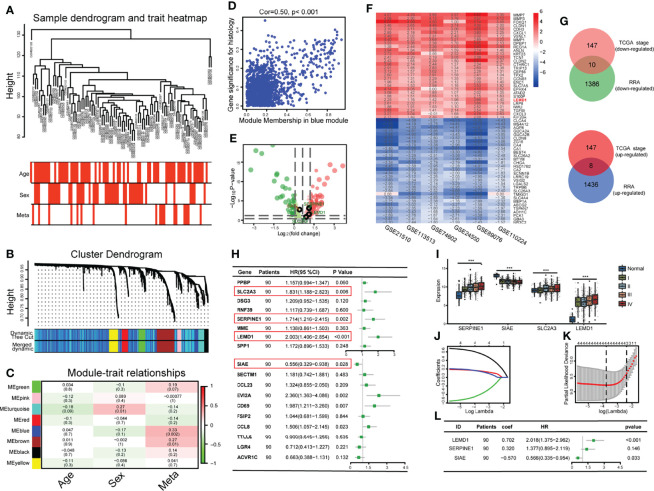
Development of an early relapse model: **(A)** Establishment of a weighted co-expression network and identification of core modules; **(B)** Gene clustering and identification of gene modules; **(C)** Correlation between gene modules and clinical data; **(D)** A scatterplot of Gene Significance (GS) vs. Module Membership (MM) in the blue module.; **(E)** Volcano map of differentially expressed genes between stages I/II and III/IV. Green dots indicate lowly expressed genes in stage III/IV. Red dots indicate highly expressed genes. **(F)** Heat map of the 30 most robust differentially expressed genes in the 6 CRC datasets analyzed by RRA analysis; **(G)** 18 Hub genes were obtained through the intersection of the genes selected by the RRA method and the WGCNA method; **(H)** Hub genes were subjected to univariate regression analysis and forest plots were drawn; **(I)** The expression of SLC2A3, SERPINE1, LEMD1, and SIAE are related to the TNM stage; **(J, K)** The Lasso regression model analysis was designed to exclude highly correlated (co-expressed) genes and to prevent the model from overfitting; **(L)** Multivariate regression analysis Forest Map consisting of SERPINE1, LEMD1, and SIAE. ***P < 0.001.

We constructed a WGCNA network using 4699 genes screened from the GSE33113 dataset. As shown in **Figures 2A, B**, based on the number of genes in the defined module, we obtained eight enriched gene modules. Further correlation analysis (**Figure 2C**, Cor = 0.33, p = 0.002) revealed that the blue module was significantly associated with tumor metastasis. Finally, we plot a scatterplot of Gene Signicance vs. Module Membership in the blue modules (**Figure 2D**).

We then divided the 1339 genes in the blue module into two groups based on the TNM stage (stage I/II 332; III/IV 267) in the TCGA database and obtained 312 genes differentially expressed in stage I/II and stage III/IV (SET1), which may play vital roles in CRC metastasis (**Figure 2E**).

To further screen for hub genes, we selected six CRC datasets from the GEO database (**Table 1** and **Figure 2F**). First, we separately calculated the differentially expressed genes (DEGs) between cancer and normal tissue in each data set (**Figure S1**,**Table 1** and **DATA S2**). Then, the genes with the most comprehensive differences by RRA analysis were selected (1444 up-regulated genes and 1396 down-regulated genes) (log | FC |> 0.6, p <0.05), and was named SET2. Finally, 18 genes were obtained by the intersection of SET1 and SET2 (8 tumor promoter genes, 10 tumor suppressor genes) (**Figure 2G**).

**Table 1:** Information of six gene datasets in the GEO database.

GEO	Platform	Normal	Tumor
GSE21510 (54)	GPL570	25	123
GSE113513 (55)	GPL15207	14	14
GSE74602 (56)	GPL6104	30	30
GSE24550 (57)	GPL5175	13	77
GSE89076 (58)	GPL16699	40	40
GSE110224 (59)	GPL570	17	17

### Construction of a CRC Relapse Model

To construct a CRC recurrence model, we first performed a univariate COX regression analysis based on the 18 Hub genes (**Figure 2H**), of which 4 genes (SLC2A3, SERPINE1, LEMD1, and SIAE) were correlated with postoperative recurrence (p < 0.05). The expression of these 4 genes was significantly correlated with the TNM stage (p < 0.001, **Figure 2I**). Furthermore, we performed a COX/LASSO regression analysis based on 4 genes (**Figures 2J, K**) and finally constructed the recurrence model consisting of LEMD1, SERPINE1, and SIAE as follows: Risk score = 0.702 × LEMD1 + 0.320 × SERPINE1 - 0.570 × SIAE (**Figure 2L**).

**Figure 3:**
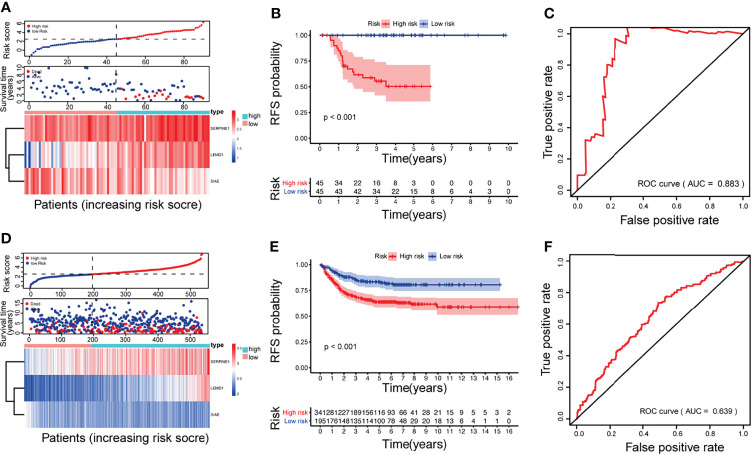
Construction and validation of the CRC relapse model: The multigene signature was based on GSE33113 **(A–C)** and validated in GSE39582 **(D–F)**. **(A, D)** survival time distribution of CRC patients and gene risk heat maps; **(B, E)** Kaplan–Meier survival analysis between patients at low- and high-risk of relapse; **(C, F)** ROC curves at 5 years.

According to the gene expression and risk coefficient of the model, each CRC patient in GSE33113 received a corresponding risk score (**Figure 3A**). The patients were then classified into high- and low-risk groups according to the median gene expression (Median = 2.4669). Kaplan–Meier plot analysis showed that patients in the high-risk group have a higher recurrence rate (**Figure 3B**, p <0.01). The ROC curve further evaluated the 5-year recurrence efficiency (**Figure 3C**). The area under the curve (AUC) was 0.883. The prognostic accuracy was validated in the GSE39582 and TCGA databases (**Figures 3D–F**: p_GSE39582_ <0.001, AUC_GSE39582_ = 0.639; **Figure S2A–C**: p_TCGA_ <0.001, AUC_TCGA_ = 0.648).

### Combined With Clinicopathological Characteristics

The relationship between the recurrence model and the clinicopathological characteristics of CRC patients, including age, sex, AJCC TNM, pathological differentiation, and the KRAS/BRAF mutation, was evaluated. As shown in **Table 2** and **Table S1**, the risk score was positively correlated with TNM stage, KRAS/BRAF mutations, vascular invasion, and CEA expression.

**Table 2:** Correlations between relapse model and clinicopathological characteristics in the GSE39582 dataset.

Characteristics	Risk score of models		Cor	P-value
	Low-risk	High-risk		
Age			0.006	0.886
<60	51	87		
≥60	144	253		
Sex			0.037	0.398
Male	92	148		
Female	103	193		
AJCC stage			0.159	<0.001
I	21	16		
II	101	159		
III	67	138		
IV	4	26		
BRAF/KRAS mutation			0.204	<0.001
0	106	153		
1	54	140		
2	12	35		
Chemotherapy			0.073	0.090
No	121	187		
Yes	73	154		
Location			0.073	0.090
Distal	133	186		
Proximal	62	155		

0: no mutation, 1: BRAF or KRAS mutation, 2: BRAF and KRAS mutation.

**Figure 4:**
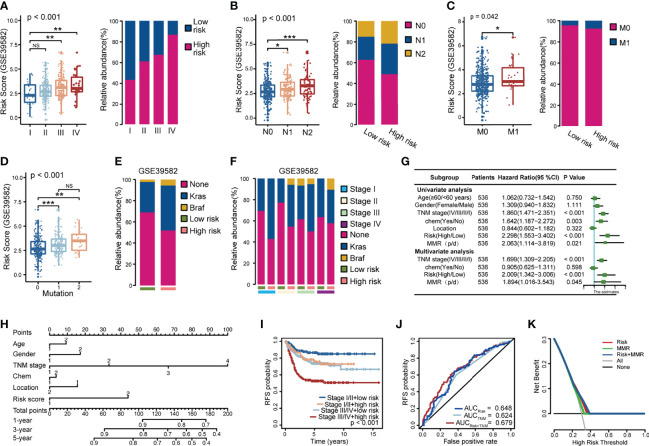
Correlation between the model and clinicopathological characteristics in the GSE39582 dataset. **(A–C)** Correlation between risk score and TNM stage **(A)**, lymphatic metastasis **(B)**, distant metastasis **(C)**; **(D-F)** Relationship between risk score and KRAS and BRAF tumor mutations, 0: None mutation: 1: KRAS mutation; 2: BRAF mutation; **(G)** Univariate and multivariate COX regression models showed that the relapse model was an independent prognostic factor; **(H)** Relapse risk nomogram based on clinical characteristics; **(I)** The combined tumor stage and risk score could effectively assess early relapse; **(J)** The ROC curve assessed the prognostic accuracy of the TNM staging and the risk score; **(K)** DCA analysis for risk score, MMR and the two combined model. NS, Not Significant; *p < 0.05; **p < 0.01; ***p < 0.001.

Furthermore, with increasing TNM stage, the risk score, and the ratio of patients with high scores also increased significantly (**Figure 4A** and **Figure S2D**). Patients with lymph node metastases (**Figures 4B** and **Figure S2E**) or distant metastases (**Figures 4C** and **Figure S2F**) had higher risk scores.

CRC metastasis is influenced by multiple molecules and is closely related to EGFR. As shown in **Figures 4D–F**, patients with KRAS/BRAF mutations have a significantly higher risk score. Thus, CRC patients with high-risk scores had a visibly higher probability of KRAS and BRAF mutations, and this phenomenon was particularly evident in patients with TNM stages I, II, and III.

After multivariate analysis adjusted for clinicopathological variables, the model was a powerful and independent prognostic factor for CRC in GSE35982 (**Figure 4G**). Furthermore, we constructed a nomogram based on the contribution of each influencing factor to the prognosis and obtained the total score of the patient. The probability of recurrence of each patient was predicted at 1, 3, and 5 years (**Figure 4H**).

By combining the TNM stage and the risk score, 536 CRC patients were classified into four groups. Patients with stage III/IV + high-risk score had a significantly higher risk of relapse, while patients with stage I/II + low-risk score had a better prognosis (**Figure 4I**, **Figure S2G**). The integrated signature of the TNM stage and the risk score to predict early relapse was superior to the TNM stage and the risk score alone at 5 years (AUC = 0.679) (**Figure 4J**).

Patients who received adjuvant chemotherapy had short recurrence-free survival times in the univariate COX survival analysis, implying that some patients did not benefit from chemotherapy. The current guidelines are equivocal for indications relative to adjuvant chemotherapy for patients with stage II tumors having poor prognostic characteristics. To validate the value of our model, we performed Kaplan–Meier analysis in patients with TNM stage II who had not received any preoperative adjuvant therapy using the GSE35986 dataset. Patients with stage II tumors showed a lower tendency for relapse in the low-risk score group (**Figure S2H**). We evaluated the risk score and mismatch repair (MMR) genes in stage II patients using decision curve analysis (DCA) (**Figure 4K**) and found that the model performed better to predict RFS. Integration of the risk score and the MMR added greater diagnostic value and provided guidance for adjuvant treatment of stage II patients.

### LEMD1 Is Highly Expressed in CRC and Is Associated With Postoperative Recurrence

**Figure 5:**
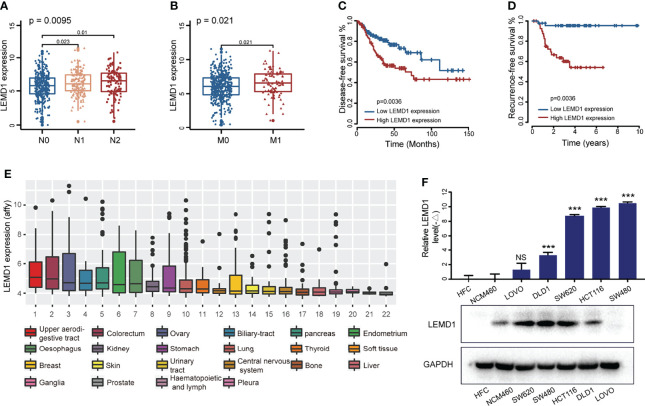
LEMD1 was abnormally expressed in CRC and was related to the prognosis of CRC patients. LEMD1 expression in CRC tissue in the TCGA database according to different N stage **(A)** and M stage **(B–D)** patients with low expression of LEMD1 have shorter disease-free survival **(C)** and lower risk of postoperative recurrence **(D, E)** LEMD1 expression in 22 cancer cell lines in the CCLE database; **(F)** LEMD1 mRNA and protein expressions in five CRC cell lines and two intestinal epithelial cells. NS, Not Significant; ***p < 0.001.

The model constructed by LEMD1, SERPINE1, and SIAE could easily predict the risk of postoperative recurrence, implying that these hub genes may play a key role in tumor occurrence and development. Of these three genes, LEMD1 had the highest ranked risk factor and was the least studied in colon cancer. Therefore, we performed further research. LEMD1 was significantly upregulated in CRC tumor tissues (p <0.001) (**Figure S3A**) and was highly expressed in lymph node metastasis and distant metastatic CRC tissues (**Figures 5A, B**), although there were no significant differences across different T stages (**Figure S3B**). Kaplan–Meier survival analysis suggested that CRC patients with high expression of LEMD1 had worse overall survival (**Figure S3C**), shorter disease-free survival (**Figure 5C**) and a higher risk of postoperative recurrence (**Figure 5D**). The above-mentioned results further indicated that LEMD1 might play a crucial role in CRC carcinogenesis.

### LEMD1 Promoted CRC Cell Invasion and Migration

LEMD1 expression was detected in the CCLE database (https://portals.broadinstitute.org/ccle) and the GSE97023 dataset. As shown in **Figure 5E**, LEMD1 was highly expressed in CRC cells among 22 common tumor cells (1060 strains), and the highest expression was found in SW480 (**Figure S3D**). Additionally, LEMD1 expression was detected in five CRC cells (DLD1, LOVO, SW620, HCT116, and SW480) and two normal intestinal epithelial cells (HFC and NCM460) using a PCR and Western blotting assays (**Figures 5F**). Compared to normal intestinal epithelial cells, LEMD1 showed higher expression in HCT116 and SW480 cells (p <0.05).

**Figure 6:**
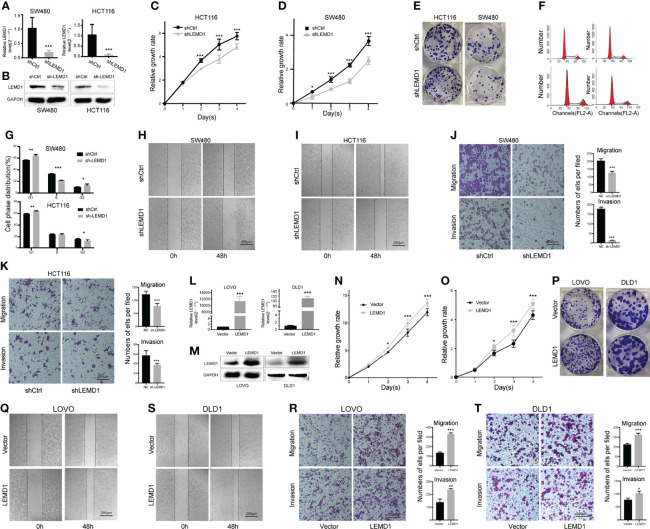
Effects of LEMD1 on the biological behavior of CRC cells. **(A, B)** LEMD1 knockdown in SW480 and HCT116 was confirmed by qRT-PCR **(A)** and western blotting **(B)**; LEMD1 knockdown suppressed SW480 **(C)** and HCT116 **(D)** proliferation determined by the CCK8 assay and clone formation assay **(E)**; **(F, G)** Cell cycle changes after knockdown LEMD1; **(H, I)** LEMD1 knockdown inhibited cell migration by the wound healing test; **(J, K)** LEMD1 knockdown inhibited invasion and migration of SW480 **(J)** and HCT116 **(K)** cells by the Transwell assay; **(L, M)** LEMD1 overexpression in LOVO and DLD1 was confirmed by qRT-PCR **(L)** and western blotting **(M)**; LEMD1 promoted proliferation of LOVO **(N)** and DLD1 **(O)** as determined by CCK8 assay and clone formation assay **(P)**; Overexpression LEMD1 promoted cell migration by the wound healing test **(Q, S)**; Overexpression LEMD1 promoted LOVO **(R)** and DLD1 (T) cell invasion and migration by the Transwell assay. *p < 0.05; **p < 0.01; ***p < 0.001.

To verify the biological function of LEMD1, we knocked down LEMD1 expression in the HCT116 and SW480 cell lines using interfering lentivirus. PCR and Western blotting analysis confirmed the efficiency of the knockdown (**Figures 6A, B**). CCK8 experiments and clone formation experiments demonstrated that LEMD1 knockdown could weaken the proliferation and colony formation abilities of SW480 and HCT116 cells (**Figures 6C–E**). The cell cycle findings indicated that SW480 and HCT116 cells were arrested in the G1 phase after LEMD1 knockdown (**Figures 6F, G**).

We further performed Transwell cell migration/invasion experiments (in Matrigel coated wells) and wound healing experiments. Compared to the control group, the migration/invasiveness of HCT116 and SW480 cells was dramatically reduced in the shLEMD1 group (p <0.001) (**Figures 6H–K**).

We constructed a LEMD1 overexpression lentivirus vector GV492 to be transfected in both LOVO and DLD1 cell lines. The overexpression efficiency was confirmed by qRT-PCR and Western blotting assay (**Figures 6L, M**). Overexpression of LEMD1 enhanced the proliferation capacity of LOVO and DLD1 cells (**Figures 6N–P**). Transwell experiments and wound healing assays showed that the invasive and migratory abilities LOVO (**Figures 6Q, R**) and DLD1 (**Figures 6S, T**) were markedly increased after LEMD1 overexpression.

### LEMD1 Promoted the Epithelial-Mesenchymal Transition

**Figure 7:**
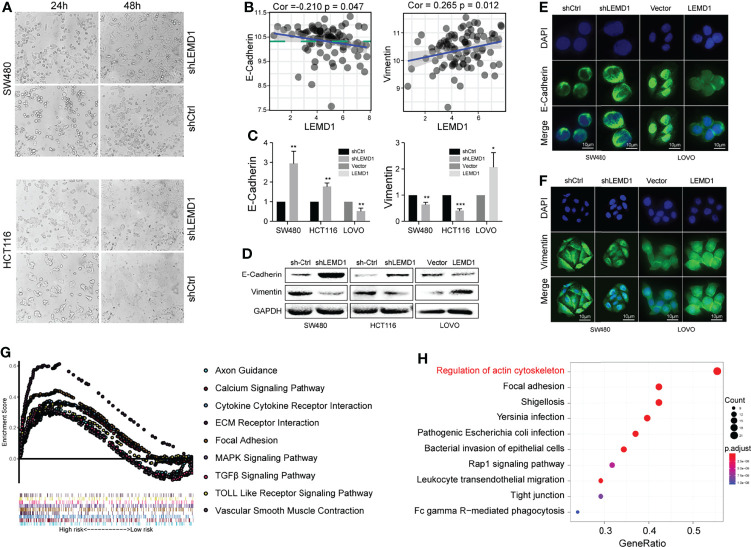
LEMD1 promotes the epithelial-mesenchymal transition. **(A)** Knockdown of LEMD1 expression changed the morphology of SW480 and HCT116 after 24 and 48 h cultures; **(B)** Correlation between LEMD1 and EMT biomarkers in GSE33113; **(C, D)** qRT-PCR **(C)** and western blotting **(D)** to detect E-cadherin and vimentin in cells overexpressed with shLEMD1 and LEMD1-overexpressed cells; Immunofluorescence to detect E-cadherin **(E)** and vimentin **(F)** after LEMD1 knockdown and overexpression in SW480 and LOVO; **(G)** GSEA enrichment analysis of LEMD1-related pathways in the TCGA database; **(H)** KEGG enrichment analysis revealed the pathway related to LEMD1. *p < 0.05; **p < 0.01; ***p < 0.001.

EMT is a common phenomenon that occurs during tumor metastasis of diverse types of cancer, including CRC. As shown in **Figure 7A**, LEMD1 knockdown significantly influenced the shrinkage morphology of the cell. The results of the correlation analysis showed that LEMD1 was negatively correlated with E-cadherin (Cor_E-cad_ = -0.210, p_E-cad_ = 0.047) and positively correlated with vimentin (Cor_vim_ = 0.265, p_vim_ = 0.012) expression in the GSE33113 dataset (**Figure 7B**). Knockdown of LEMD1 in HCT116 and SW480 could increase the expression of E-cadherin and decrease the expression of vimentin, whereas the overexpression of LEMD1 in LOVO showed opposite changes (**Figures 7C, D**). Comparable results were observed with the immunofluorescence assay (**Figures 7E, F**). The results mentioned above indicated that LEMD1 could promote the EMT process in CRC cells.

### LEMD1 Promoted CRC Metastasis *via* the RhoA/ROCK Pathway

GSEA enrichment analysis was performed and revealed the LEMD1-related pathways (p <0.05, **Figure 7G**). Furthermore, we used the Utility RT Profiler PCR Array to detect regulated genes after knockdown/overexpression of LEMD1. As shown in **Figure S4A**, we obtained 13 genes that were positively related to LEMD1 and 32 genes that were negatively related. Further KEGG pathway and GO analysis was applied to analyze differentially expressed mRNAs. Pathway analysis indicated that the most significant pathways were those that regulated the actin cytoskeleton and cell adhesion (**Figure 7H**). The most significant biological processes, cellular components, and molecular function as indicated by GO analysis were the regulation of cell morphology, regulation of actin-based processes such as cell migration, actin cytoskeleton, adhesion, formation of adhesion molecules, and Rho GTPase binding (**Figure S4B–D**).

**Figure 8:**
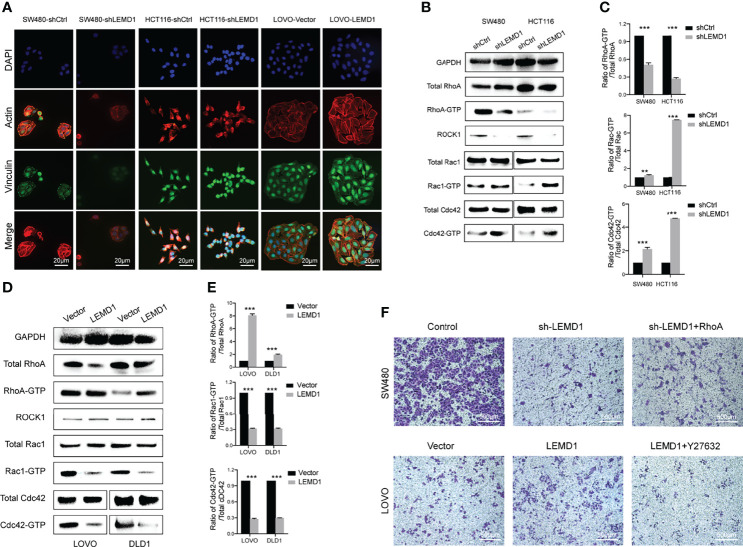
LEMD1 promotes CRC metastases through the RhoA/ROCK pathway. **(A)** Actin and Vinculin changes after knockdown/overexpression LEMD1; **(B, C)** Knockdown of LEMD1 could decrease RhoA activity while activating GTP-Rac and GTP-Cdc42; **(D, E)** Overexpression of LEMD1 could activate GTP-RhoA and decrease the activity of GTP-Rac and GTP-Cdc42; **(F)** Overexpression of RhoA partially restored the inhibitory effect of knockdown of LEMD1 on cell migration; Y27632 could reduce the effect of LEMD1 on cell migration. **p < 0.01; ***p < 0.001.

Previous studies have shown that RHOA and the Rho GTPase family (Rac and Cdc42) play key roles in the cytoskeleton properties including, cell adhesion, and cell migration by converting GDP binding or GTP binding. We used immunofluorescence assays to observe changes in actin and vinculin expression after LEMD1 knockdown and overexpression. As shown in **Figure 8A**, the fluorescence intensities of actin (red) and vinculin (green) were significantly weakened after the removal of LEMD1 in SW480 and HCT116. However, they were enhanced after overexpressing LEMD1 in LOVO.

RhoA-GTP pull-down experiments were also performed to detect RhoA expression in the activated form. As shown in **Figure 8B**, RhoA-GTP levels and the RhoA-GTP/total RhoA ratio decreased significantly in the shLEMD1 group (**Figure 8C**). In contrast, RhoA-GTP levels and the RhoA-GTP/total RhoA ratio increased after LEMD1 overexpression in LOVO and DLD1 (**Figures 8D, E**). Furthermore, as a downstream effector of RhoA, ROCK1 was positively related to LEMD1.

We then examined other members of the RhoGTP family (Rac and Cdc42). In striking contrast to RhoA regulation, the knockdown of LEMD1 induced a strong activation of Rac1 and Cdc42 (**Figures 8B, C**). LEMD1 overexpression reduced Rac1 and Cdc42 protein activities in LOVO and DLD1 cells (**Figures 8D, E**).

To further determine whether the RhoA/ROCK1 signaling pathway was necessary for the oncogenicity of LEMD1, we transfected the RhoA overexpression plasmid into SW480 cells with stable knockdown of LEMD1 expression and in a control group. As shown in **Figure 8F**, sh-LEMD1 treated with a vehicle control significantly inhibited cell migration, while RhoA overexpression could partially restore the migration ability of CRC cells. Similarly, the ROCK1 inhibitor Y27632 could significantly reduce the number of migrating cells induced by LEMD1 overexpression in LOVO, indicating that LEMD1-induced migration was partially dependent on the RhoA/ROCK signaling pathway.

## Discussion

Previous studies have attempted to identify ideal molecular markers for the prediction of early relapse of CRC by constructing specific gene sets (14, 31–36), lncRNAs (37–39), miRNAs (40–45), methylation (46), metabolites (47, 48) and other models (49, 50). However, most are limited to validation in databases and exhibited finite clinical value.

In this study, we performed WGCNA analysis and RRA analysis using public datasets and constructed a CRC relapse model composed of SERPINE1, LEMD1, and SIAE. The clinical value was further validated in two independent datasets. Our model was related to tumor TNM staging, pathological staging, and KRAS/BRAF mutational status and played an auxiliary role in the diagnosis and prognosis of CRC. The combination of our model and TNM staging could predict early relapse of CRC with greater precision.

During cell migration, the anterior bulge forms an adhesion to the extracellular matrix, whereas the cell body and tail contract. Both the actin cytoskeleton and microtubules are crucial for this process. Rho GTPases belong to the Ras superfamily and are involved in many processes of tumor progression such as cell transformation, cytokinesis, angiogenesis, extracellular matrix deposition, and tumor metastasis (51). As one of the most widely studied members of the small GTPase Rho family, RhoA has been reported to mediate the contractility of actin-myosin and is involved in the formation of stress fibers (6, 52). RhoA also is involved in membrane folding, formation of plasma membrane vesicles, and stress fiber formation by affecting ROCK and mDia; it plays a role in the formation of the leading edge protrusion of cancer cells (53). Current research indicates that the activation of RhoA occurs before the activation of Rac and Cdc42 (54). Like other GTPase family members, RhoA is activated through binding to its GTP to activate downstream molecules, which are important for the cytoskeleton, cell adhesion, cell invasion, metastasis and the occurrence of EMT (55).

We used GTP pull-down and Western blot assays to detect the expression of GTP-RhoA following the knockdown/overexpression of LEMD1. The results showed that LEMD1 could upregulate the expression of GTP-RhoA and ROCK1. Therefore, we concluded that LEMD1 affected cytoskeletal changes by activating the RhoA/ROCK signaling pathway, thus promoting EMT and tumor metastasis.

Rho GTPase signaling is a complex regulatory network. RhoA, Rac1, and Cdc42 interact spatially and temporally during cancer cell migration: for example, 1-phosphosphingol (S1P) can co-stimulate cancer cell movement by binding to S1P receptors and activating Rac1 and Cdc42. Conversely, S1P regulates and inhibits the movement of cancer cells *via* a S1P receptor 2-dependent activation of RhoA (56). Therefore, the role of S1P depends on the preponderance of the expression of receptor subtypes in cancer cells. In addition, activated RhoA also inhibits Rac activation by inhibiting β-Pix recruitment to the adhesive plaques at the tail of migrating cells (57). RhoA and Cdc42 can also interact spatially and temporally during the formation of invasive pseudopods (58). RhoA plays a prominent role in the formation of invadopdia and its activation predates CDC42, while RAC plays an opposite role in this process (59). Our results suggest that LEMD1 may play a role in invadopdia formation by up-regulating GTP-RhoA. However, the specific regulatory mechanisms of LEMD1, CDC42 and RAC1 needs further study.

To investigate whether LEMD1 regulates cell metastasis depending on the RhoA signaling pathway, we co-transfected RhoA overexpression plasmids into LEMD1 stable knockdown SW480 and applied the ROCK inhibitor Y27632 to LEMD1 overexpressing LOVO cell lines. The Transwell assay was then used to detect changes in migratory capacity. The overexpression/interference of the RhoA/ROCK1 signaling pathway could partially restore/inhibit the effects of LEMD1 on the migratory capacity of CRC cells. Therefore, we believe that RhoA is a downstream regulator of LEMD1, and LEMD1 could affect cytoskeleton changes and tumor cell migration in part through the RhoA/ROCK1 signaling pathway.

## Conclusion

In this study, a CRC-relapse model composed of LEMD1, SERPINE1, and SIAE was constructed by the comprehensive analysis of multiple CRC datasets. Further *in vitro* experiments showed that LEMD1 could regulate CRC cell proliferation, migration, invasion, and promote EMT transition. Finally, we determined that LEMD1 promotes CRC cell migration through the RhoA/ROCK signaling pathway. These findings are relevant for the diagnosis and treatment of postoperative CRC relapse.

## Data Availability Statement

The datasets presented in this study can be found in online repositories. The names of the repository/repositories and accession number(s) can be found in the article/**Supplementary Material**.

## Ethics Statement

The study was carried out in accordance with the principles of the Helsinki Declaration and was approved by the Ethics Boards of Jiangsu Cancer Hospital. The patients/participants provided their written informed consent to participate in this study.

## Author Contributions

HZ designed the study and performed the experiments, analyzed the data, and wrote the manuscript. CX helped with the experiments. FJ assisted in the preparation, review, editing, and revision of the manuscript. JF validated the experimental results and their interpretation. All authors contributed to the article and approved the submitted version.

## Funding

China Postdoctoral Science Foundation (Grant No. 2021M701500).

## Conflict of Interest

The authors declare that the research was conducted in the absence of any commercial or financial relationships that could be construed as a potential conflict of interest.

## Publisher’s Note

All claims expressed in this article are solely those of the authors and do not necessarily represent those of their affiliated organizations, or those of the publisher, the editors and the reviewers. Any product that may be evaluated in this article, or claim that may be made by its manufacturer, is not guaranteed or endorsed by the publisher.

## Supplementary Material

Supplementary Figure 1 Volcano diagram of differentially expressed genes in the GSE21510, GSE113513, GSE74602, GSE24550, GSE89076, and GSE110224 datasets: red indicates upregulated genes; blue indicates downregulated genes.

Supplementary Figure 2 The relationship between the risk score and the clinicopathological data in TCGA database. **(A–C)** are recurrence models validated in the TCGA database; **(D–F)** Relationship between risk score and TNM stage **(D)**, lymphatic metastasis **(E)**, distant metastasis **(F, G)** The combined TNM stage and risk score (The risk coefficient was calculated according to the multivariate COX regression model in **Figure 4G**: 1.699 × TNM stage + 2.009× risk score) could effectively assess early relapse; **(H)** Patients with stage II tumors showed a lower tendency for relapse in the low-risk score group. **p < 0.01; ***p < 0.001.

Supplementary Figure 3 Effects of LEMD1 on the biological behavior of CRC cells. **(A)** LEMD1 expression in six CRC datasets from the GEO database; **(B)** LEMD1 expression in the TCGA database according to different T stage; **(C)** patients with low expression of LEMD1 have shorter OS; **(D)** LEMD1 expression in 34 CRC cell lines in GSE97023.

Supplementary Figure 4 Enrichment analysis revealed the pathway associated with LEMD1. The Utility RT Profiler PCR chip was used to detect changes in gene expression after LEMD1 knockdown/overexpression in SW480 and LOVO. **(A)** Taking the intersection, 12 genes were positively correlated with LEMD1, and 32 genes were negatively correlated. **(B–D)** GO enrichment analysis revealed the biological processes **(B)**, cytological components **(C)**, and molecular biological functions **(D)** in which LEMD1 may be involved.
