# Supplementary figures and images for: A Three-Genes Signature Predicting Colorectal Cancer Relapse Reveals LEMD1 Promoting CRC Cells Migration by RhoA/ROCK1 Signaling Pathway
Source: Front Oncol. 2022 May 10;12:823696. doi: 10.3389/fonc.2022.823696 (PMC9127067; doi:10.3389/fonc.2022.823696)

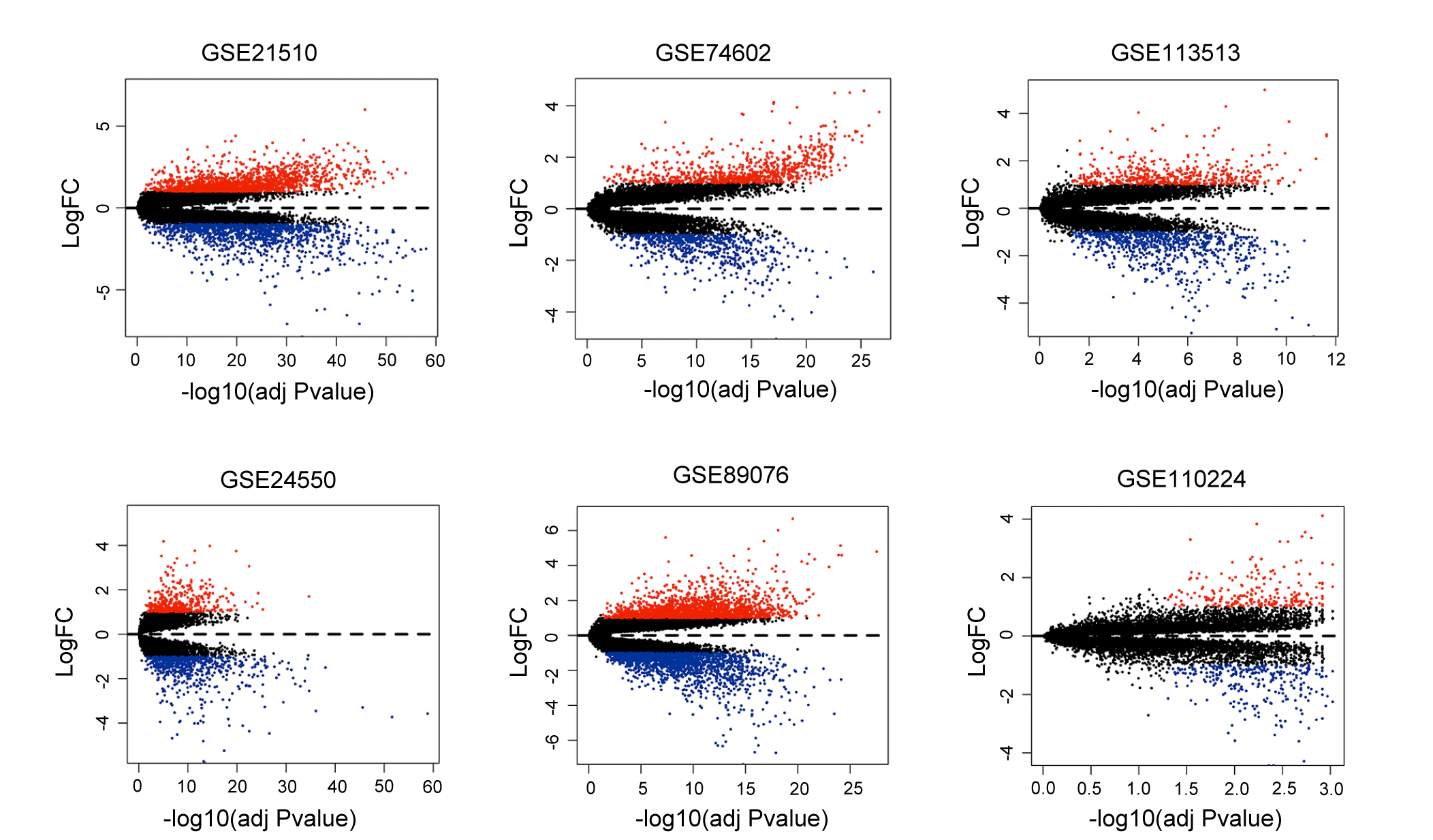

Supplement: Supplementary Figure 1 — Volcano diagram of differentially expressed genes in the GSE21510, GSE113513, GSE74602, GSE24550, GSE89076, and GSE110224 datasets: red indicates upregulated genes; blue indicates downregulated genes. [file Image_1.tif]

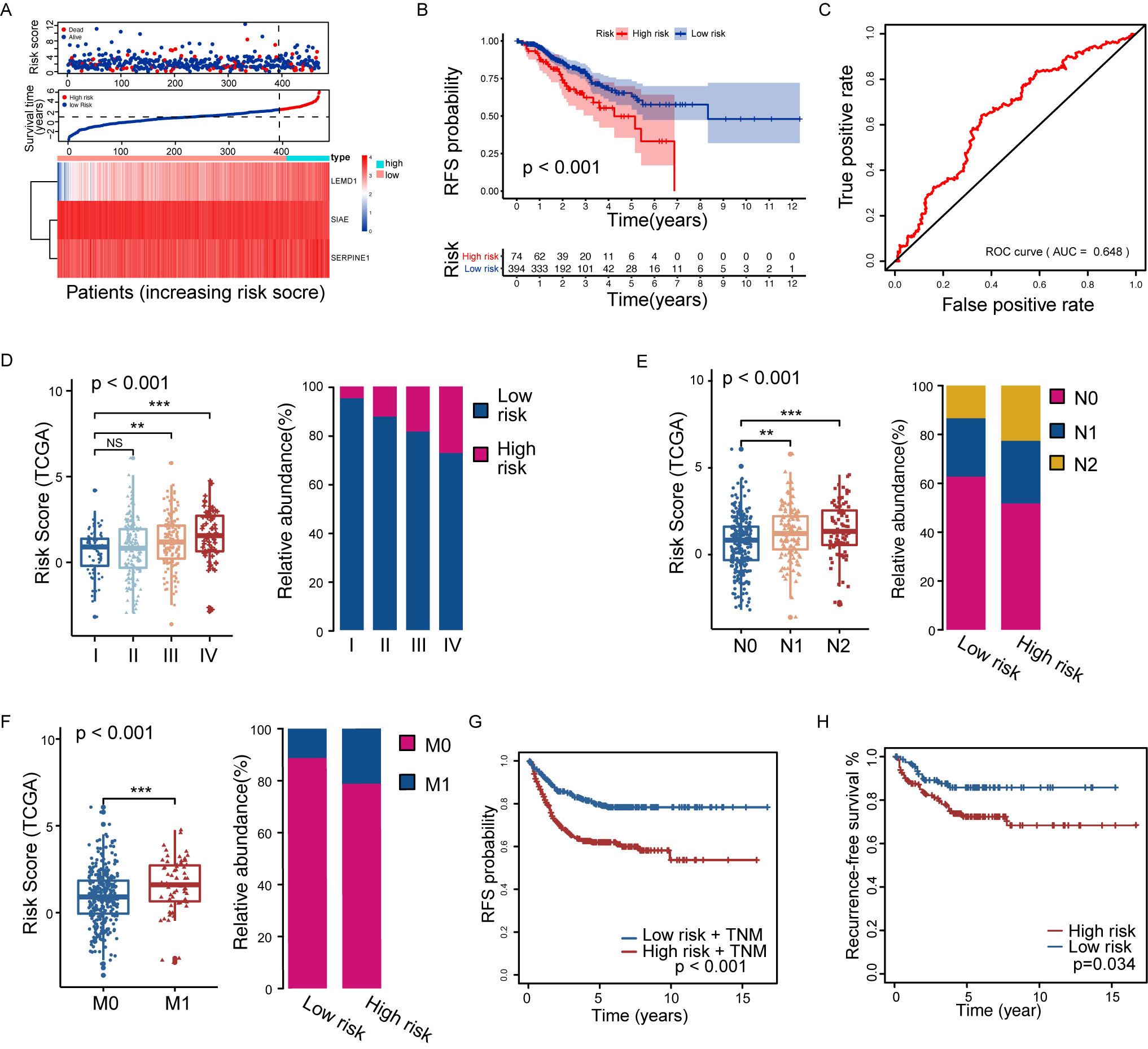

Supplement: Supplementary Figure 2 — The relationship between the risk score and the clinicopathological data in TCGA database. (A–C) are recurrence models validated in the TCGA database; (D–F) Relationship between risk score and TNM stage (D), lymphatic metastasis (E), distant metastasis (F, G) The combined TNM stage and risk score (The risk coefficient was calculated according to the multivariate COX regression model in Figure 4G: 1.699 × TNM stage + 2.009× risk score) could effectively assess early relapse; (H) Patients with stage II tumors showed a lower tendency for relapse in the low-risk score group. **p < 0.01; ***p < 0.001. [file Image_2.tif]

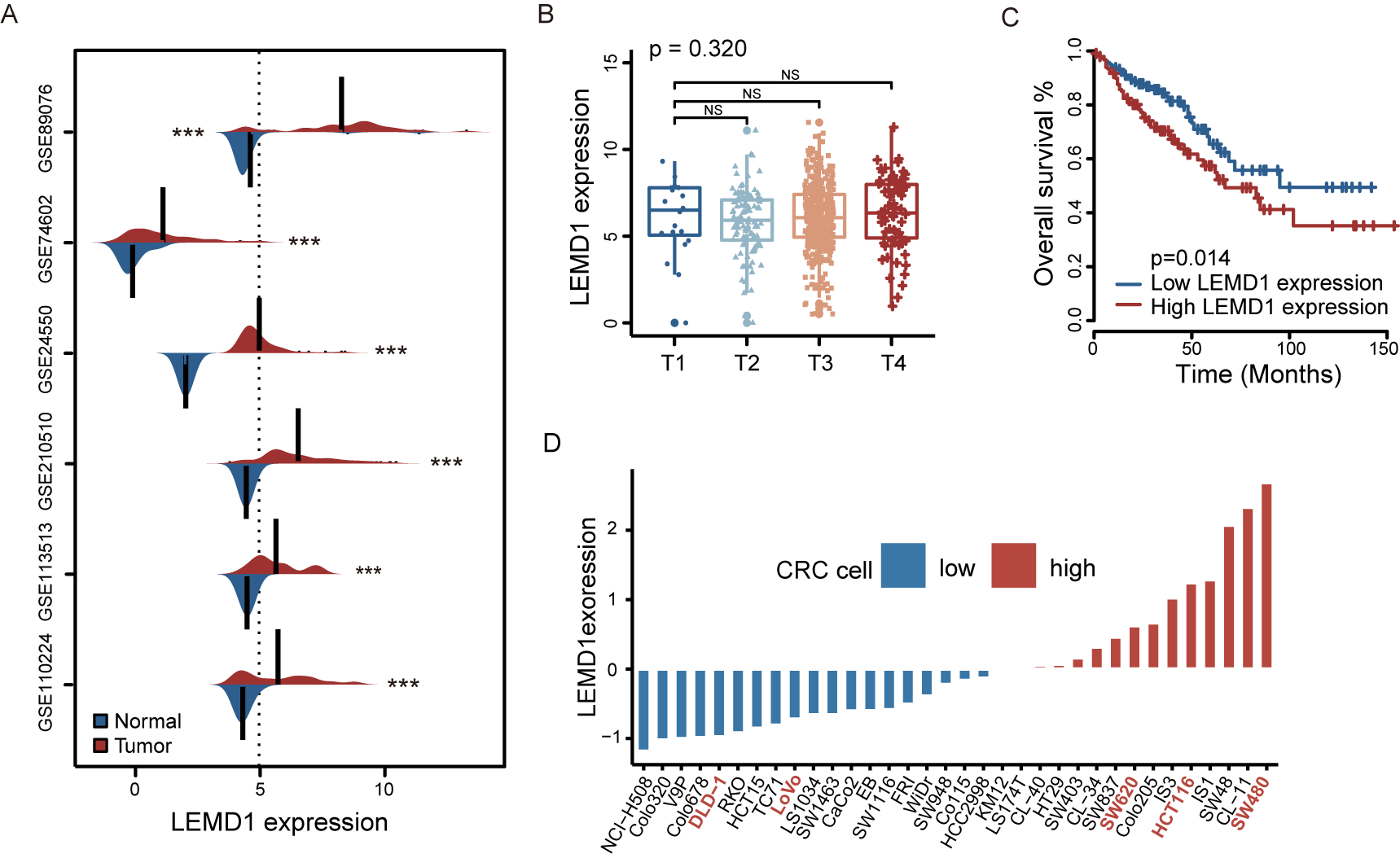

Supplement: Supplementary Figure 3 — Effects of LEMD1 on the biological behavior of CRC cells. (A) LEMD1 expression in six CRC datasets from the GEO database; (B) LEMD1 expression in the TCGA database according to different T stage; (C) patients with low expression of LEMD1 have shorter OS; (D) LEMD1 expression in 34 CRC cell lines in GSE97023. [file Image_3.tif]

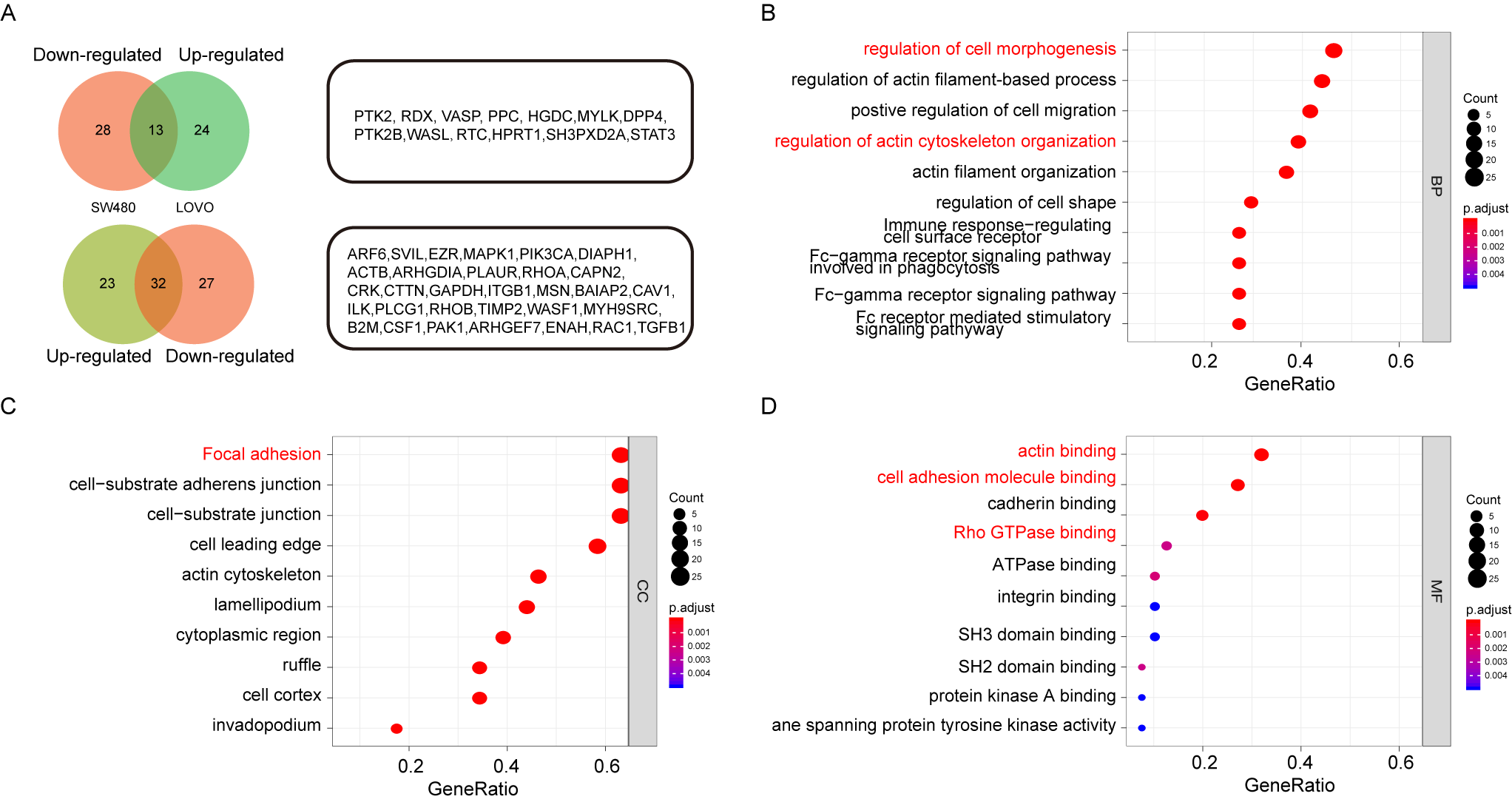

Supplement: Supplementary Figure 4 — Enrichment analysis revealed the pathway associated with LEMD1. The Utility RT Profiler PCR chip was used to detect changes in gene expression after LEMD1 knockdown/overexpression in SW480 and LOVO. (A) Taking the intersection, 12 genes were positively correlated with LEMD1, and 32 genes were negatively correlated. (B–D) GO enrichment analysis revealed the biological processes (B), cytological components (C), and molecular biological functions (D) in which LEMD1 may be involved. [file Image_4.tif]
