# Supplementary material for: A Three-Genes Signature Predicting Colorectal Cancer Relapse Reveals LEMD1 Promoting CRC Cells Migration by RhoA/ROCK1 Signaling Pathway
Source: Front Oncol. 2022 May 10;12:823696. doi: 10.3389/fonc.2022.823696 (PMC9127067; doi:10.3389/fonc.2022.823696)
Supplement: Supplementary file 5 [file DataSheet_1.docx]

The primers used were as follows.

| Gene | Sequence |
| --- | --- |
| LEMD1-forward | ATTGCAGAACCAACTTGAGAAGC |
| LEMD1-reverse | CGCGCAGTAGTCTCTCTCTT |
| E-cadherin-forward | GAGTGAAGCCTTGAGTGC |
| E-cadherin- reverse | TTCACATCCAGCACATCC |
| Vimentin-forward | GGCTCAGATTCAGGAACAGC |
| Vimentin-reverse  GAPDH-forward  GAPDH-reverse  SERPINE1-forward  SERPINE1-reverse  SIAE-forward  SIAE-reverse | GTTGTGGGACCTGTGGAAGT   \| TGGAAGACAGAATGGAAGAA \| \| --- \| \| GCAGAGAAGCAGACAGTT \|   ACCGCAACGTGGTTTTCTCA  TTGAATCCCATAGCTGCTTGAAT  ATATGGGGCTTCGGTACACCT  TCCACGTATCAGAGTGAGCTT |
