# Supplementary material for: A Three-Genes Signature Predicting Colorectal Cancer Relapse Reveals LEMD1 Promoting CRC Cells Migration by RhoA/ROCK1 Signaling Pathway
Source: Front Oncol. 2022 May 10;12:823696. doi: 10.3389/fonc.2022.823696 (PMC9127067; doi:10.3389/fonc.2022.823696)
Supplement: Supplementary file 7 [file Table_1.docx]

**Table.S1. Correlations between relapse model and clinicopathological characteristics in TCGA database.**

| Charcteristics | Risk score of model | | Cor | P value |
| --- | --- | --- | --- | --- |
|  | Low risk | High risk |  |  |
| Age |  |  | 0.002 | 0.959 |
| <60 | 129 | 24 |  |  |
| ≥60 | 265 | 50 |  |  |
| Gender |  |  | 0.035 | 0.447 |
| Male | 184 | 31 |  |  |
| Female | 210 | 43 |  |  |
| AJCC stage |  |  | 0.222 | 0 |
| I | 80 | 3 |  |  |
| II | 141 | 22 |  |  |
| III | 114 | 26 |  |  |
| IV | 45 | 21 |  |  |
| History of polyps |  |  | 0.05 | 0.32 |
| No | 240 | 36 |  |  |
| Yes | 104 | 21 |  |  |
| Venus invasion |  |  | 0.112 | 0.021 |
| Yes | 275 | 42 |  |  |
| No | 82 | 24 |  |  |
| Residual tumor |  |  | 0.063 | 0.216 |
| R0 | 316 | 46 |  |  |
| R1 | 5 | 0 |  |  |
| R2 | 16 | 5 |  |  |
| CEA |  |  | 0.125 | 0.024 |
| Low | 143 | 19 |  |  |
| High | 128 | 34 |  |  |

CEA is grouped according to the median value
